# Modeling the heating and cooling of a chromophore after photoexcitation†

**DOI:** 10.1039/d2cp00686c

**Published:** 2022-04-05

**Authors:** Elizete Ventura, Silmar Andrade do Monte, Mariana T. do Casal, Max Pinheiro, Josene Maria Toldo, Mario Barbatti

**Affiliations:** Universidade Federal da Paraíba 58059-900, João Pessoa-PB Brazil elizete@quimica.ufpb.br silmar@quimica.ufpb.br; Aix Marseille University, CNRS, ICR Marseille France mario.barbatti@univ-amu.fr barbatti.org; Institut Universitaire de France 75231 Paris France

## Abstract

The heating of a chromophore due to internal conversion and its cooling down due to energy dissipation to the solvent are crucial phenomena to characterize molecular photoprocesses. In this work, we simulated the *ab initio* nonadiabatic dynamics of cytosine, a prototypical chromophore undergoing ultrafast internal conversion, in three solvents—argon matrix, benzene, and water—spanning an extensive range of interactions. We implemented an analytical energy-transfer model to analyze these data and extract heating and cooling times. The model accounts for nonadiabatic effects, and excited- and ground-state energy transfer, and can analyze data from any dataset containing kinetic energy as a function of time. Cytosine heats up in the subpicosecond scale and cools down within 25, 4, and 1.3 ps in argon, benzene, and water, respectively. The time constants reveal that a significant fraction of the benzene and water heating occurs while cytosine is still electronically excited.

## Abstract 2

An analytical energy-transfer model is implemented to obtain a chromophore's heating and cooling times in a given solvent by using quantities available in nonadiabatic dynamics simulations.

## Graphical abstract

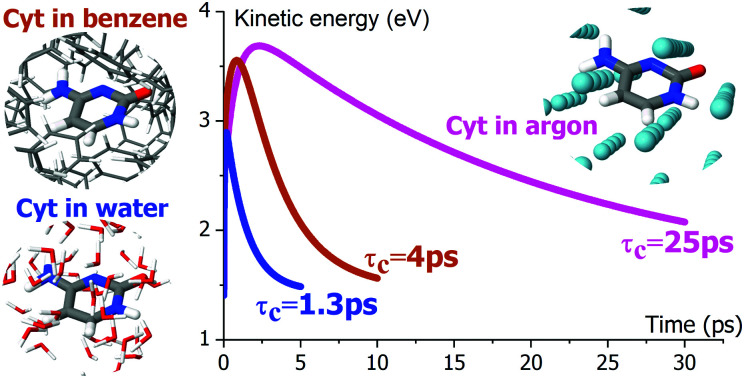

## Introduction

Photoexcitation instantaneously deposits tens of kcal mol^−1^ of energy into a chromophore. On a scale ranging from hundreds of femtoseconds to a few nanoseconds, this energy excess may induce chemical transformations, be reemitted as light, or be dissipated as heat. In the last case, there are usually two complementary (but sometimes competing) processes. First, the chromophore's vibrational degrees are heated up after the internal conversion. Second, the heat is transferred to the solvent, cooling down the chromophore. On which time scales do these heating and cooling processes occur? Naturally, we expected the answer to this question to be intensely dependent on the solvent's nature. The cooling down is restricted to slow infrared irradiation in the vacuum limit, but it may be much faster if the chromophore is firmly bound to the solvent.

**Fig. 1:**
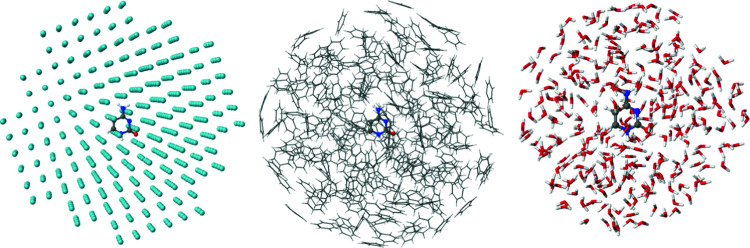
Systems studied in this work: clusters of (a) cytosine and Ar atoms; (b) cytosine and benzene molecules, and (c) cytosine and water molecules.

Our present work aims to quantify the time scales for both processes, including excited-state energy transfer and nonadiabatic events. Taking cytosine as a prototypic chromophore undergoing ultrafast internal conversion, we simulated its nonadiabatic dynamics in an argon matrix, benzene, and water (Fig. 1); thus, we gauged the energy transfer from weak van der Waals’ interactions, through medium intensity π–π interactions, to strong hydrogen bonds. Then, we proposed an analytical model to quantify the transfer rates, providing a protocol that can be used with data from any other dynamics simulations.

Not unexpectedly, we are not the first group to address the fundamental physical–chemical problem of heat transfer between a molecule and its solvent.^1–10^ Nevertheless, studies that describe solute heating and cooling in solution after an initial electronic excitation are rare.^1^ Most theoretical models rely on classical dynamics and only describe solute cooling. The cooling process is investigated in these models, supposing that the solute initially has high kinetic energy^2–6^ or high potential energy.^7,8^ In some models used to study intramolecular vibrational relaxation, the photon energy is assumed to be instantaneously converted into the vibrational energy of a given moiety.^9,10^ Recently, Balevičius Jr *et al.*^1^ proposed an approach in which a simplified Hamiltonian is used to describe both heating and cooling processes. Their model, however, does not take into account nonadiabatic and leaking effects. This latter includes the possibility that the initial excess (electronic) energy relaxes simultaneously to the internal degrees of freedom of the solute and solvent.

Our motivation to develop this project has been connected to our latest works on the development of molecular heaters for agriculture in extreme weather.^11^ Nevertheless, the range of interest of our results extends much beyond this topic. Recent technological applications profit from chromophore-solvent heat transfer, such as photothermal therapy (where the heat generated by the photoexcited chromophore is used to kill cancer cells and pathogens)^12,13^ and molecular solar thermal energy storage (where a high-energy metastable isomer can release the stored energy to the environment as heat).^14,15^ In contrast, heat release to the environment is undesirable in many other applications, such as singlet oxygen generation in photodynamic therapy,^14,16,17^ data storage,^18^ and solar light-harvesting applications.^19,20^ Additionally, the study of chromophore-solvent heat transfer is essential to understand the reactivity of molecules in solution, as the rates of these reactions can be strongly dependent upon the internal energy of the reactants and hence upon the energy flow between solute and solvent molecules.^21^ Indeed, in the presence of competing processes, the chromophore-solvent heat-transfer rates can be decisive, controlling the reaction yields.

DNA nucleobases and nucleosides are excellent models for studying the vibrational energy flow from photoexcited molecules to their surroundings because their ultrafast and complete internal conversion to S_0_.^22–24^ We have chosen cytosine as a prototypical chromophore because its internal conversion is well-characterized theoretically^25–40^ and experimentally.^31,33,39–44^ After light absorption, its excited-state dynamics is governed by a branching between competing decay channels, associated with three conical intersections, ππ*/S_0_ (ethylenic), *n*_O_π*/S_0_, and *n*_N_π*/S_0_. Although there is some debate about which one of the conical intersections dominates the nonradiative decay in the gas phase, several theoretical^25,37,45,46^ and experimental works^43,47,48^ agree on reporting ultrafast time constants (*τ*_1_ = 0.01–0.2 ps and *τ*_2_ = 0.5–2 ps), primarily associated with the decay through one of the nπ*/S_0_ conical intersections. The ultrafast nonradiative decay is connected with its photostability and effectively dissipates the harmful electronic excitation into heat.^34^

Although there are systems that show intermolecular vibrational relaxation mainly in the excited states (like perylene in ketone solvents^49^), the ultrafast internal conversion of cytosine implies a competition between relaxation from the excited and ground states. From the theoretical point of view, describing such a competition requires multiconfigurational methods to account for the nonadiabatic dynamics to the ground state.^50^ The approach used in this work (employing surface hopping simulations coupled with hybrid CASSCF/molecular-mechanics electronic structure^51,52^) can describe the relaxation independently of its time scale and source state (ground or excited). Thus, it can deal with the nonadiabatic relaxation of the initial electronic energy to the internal degrees of freedom of the solute and solvent of a realistic system, features that are not considered in most of previous approaches.

## Modeling the heat transfer

We modeled the heat transfer processes by monitoring the time evolution of mean kinetic energy during the dynamics.^53^ This evolution should have two main contributions. First, the chromophore's kinetic energy (*E*_c_) should increase after the internal conversion. Second, it should later reduce because of the energy transfer to the solvent, whose kinetic energy, *E*_s_, should correspondingly increase. Inspired by the model proposed in ref. 1, these two contributions give rise to the following phenomenological equation1
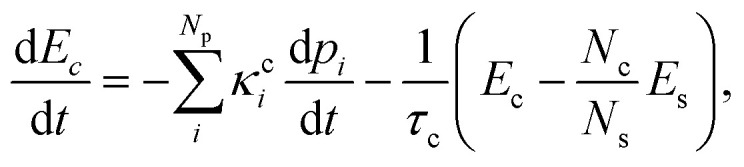
where *κ*^c^_*i*_ and *τ*_c_ are adjustable parameters, while *p*_*i*_ is the population of each excited-state reaction pathway feeding the ground state *via* internal conversion. Thus, the term proportional to d*p*_*i*_/d*t* contributes to the chromophore's heating through population transfer from the excited state. Note that each *p*_*i*_ does not correspond to different excited-state populations but – we emphasize – to different pathways. They are obtained by fitting the ground-state population (*p*_0_) with the function2
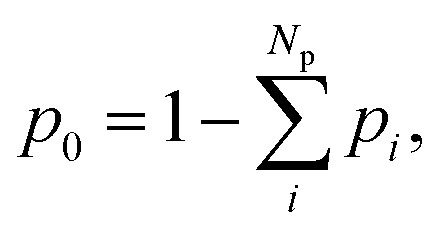
where3
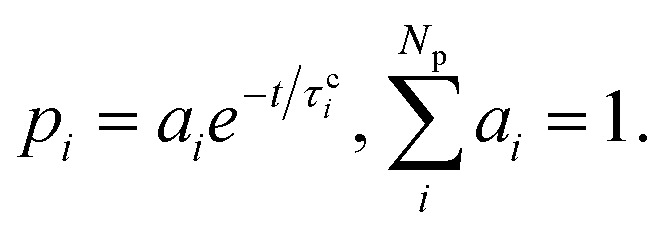


The different pathways described by each *p*_*i*_ connect the first excited state to the ground state through different time constants *τ*^c^_*i*_. In eqn (1), all three solvents could be described by two reaction pathways (*N*_p_ = 2).

The term in the parentheses on the right side of eqn (1) transfers energy to the solvent (for *E*_c_ > *N*_c_*E*_s_/*N*_s_). It is the difference between the chromophore's kinetic energy and the solvent's mean kinetic energy over the same number of atoms. (This term is analogous to what we expect from thermal contact between a hot and a cold body.) *τ*_C_ is the chromophore's cooling time constant, while *N*_c_ and *N*_s_ are the numbers of atoms in the chromophore and solvent, respectively.

In principle, *E*_s_ should evolve according to a differential equation coupled to eqn (1). However, if we assume that there is little energy back transfer from the solvent to the chromophore, we can first find out the functional dependence of *E*_s_ on time and use it to solve eqn (1). Our simulations show that4
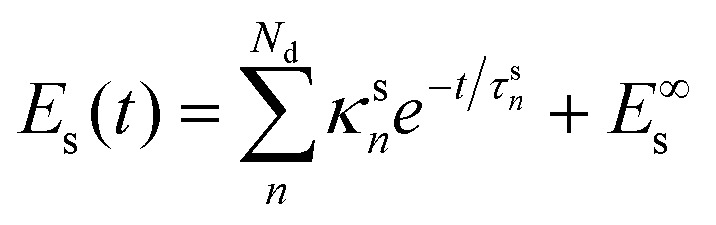
describes the solvent's kinetic energy well, where *κ*^s^_*n*_ and *τ*^s^_*n*_ are adjustable parameters. Each *τ*^s^_*n*_ is a time constant of a single solvent heating channel and *κ*^s^_*n*_ is subject to the constraint5
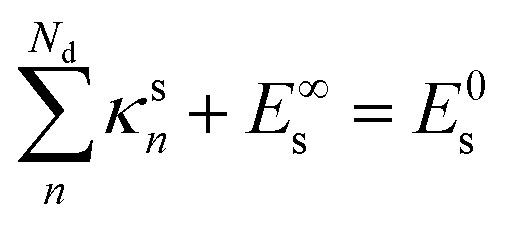
*E*^∞^_s_ is the equilibrium value of *E*_s_ and *E*^0^_s_ = *E*_s_ (0). For all solvents, *N*_d_ = 1 was enough to yield suitable fittings, which implies *κ*^s^_1_ = *E*^0^_s_ − *E*^∞^_s_.

Employing eqn (3) and (4), the solution of eqn (1) is6
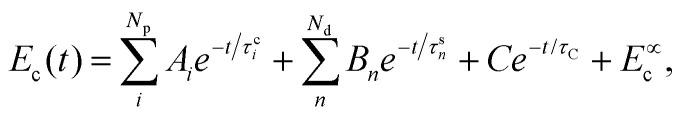
where7
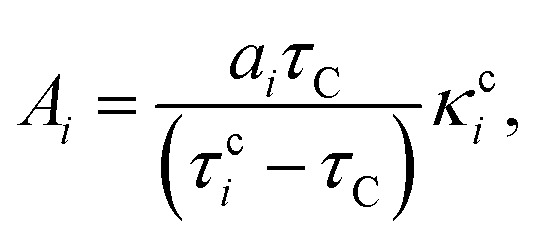
8
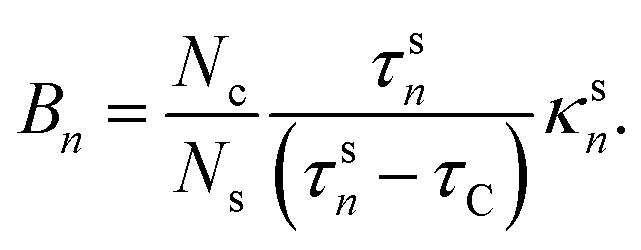


The coefficients *A*_*i*_ can either be positive or negative, reflecting the balance of heat gain or loss at that time constant. Assuming thermal equilibrium between chromophore and solvent at *t* → ∞ and that there is a harmonic virial partition between kinetic and potential energies, the asymptotic kinetic energy values of the chromophore and solvent are connected to the initial values through9
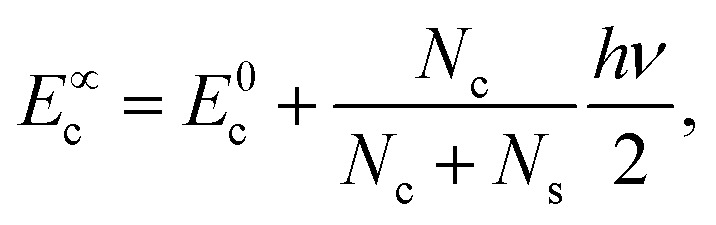
10
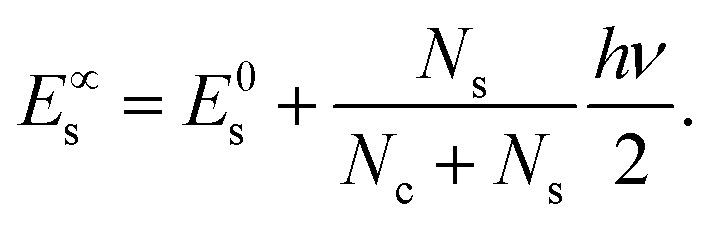


In these equations, *hν* is the photon energy, *E*^0^_c_ = *E*_c_(0), and *E*^0^_s_ = *E*_s_(0). A general discussion about these equilibrium values is in the ESI† Fig. S1.

Returning to eqn (6), *C* depends on *A*_*i*_ and *B*_*n*_ due to the boundary values of *E*_c_ at *t* = 0 and *t* → ∞:11
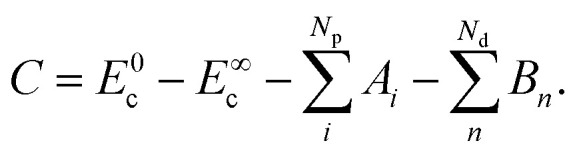


To use the model, we proceed through the following steps:

1. We fit the ground-state population from nonadiabatic dynamics simulations with eqn (2) to obtain *a*_*i*_ and *τ*^c^_*i*_;

2. We use the kinetic energy of the solvent, also coming from dynamics simulations, to determine *E*^∞^_s_ with eqn (10) and get *κ*^s^_*n*_ and *τ*^s^_*n*_ through a fitting procedure;

3. We use the kinetic energy of the chromophore to get *E*^∞^_c_ (eqn (9)) and fit these data with eqn (6) to determine *κ*^c^_*i*_ and *τ*_C_. These parameters are constrained to be ≥0.

This procedure allows determining the time to heat the chromophore due to internal conversion and the time to cool it down due to energy transfer to the solvent. The heating time is given by the mean excited-state lifetime12
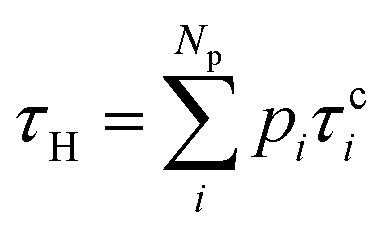
and the cooling time by *τ*_C_.

Each *τ*^s^_*n*_ in eqn (4) is a time constant measuring a single channel of solvent heating. In turn, *τ*_C_ is a single time constant describing the overall solute cooling. In a fully coupled model, these constants balance, and a common time constant given by the harmonic mean of all these time constants emerges. Our model, however, neglects this back coupling by proposing a solvent kinetic energy dependence (eqn (4)) independent of the solute. Thus, the complementarity between time constants is lost. If the solvent heats through a single channel and there is no heat *via* internal conversion, we could impose this complementarity as an additional constraint. However, we do not see how to do it when multiple channels are present. We will see in the results that neglecting the back couplings is justifiable in all three cases we examined. Thus, we focused on *τ*_C_ time constant as the measure of the solute cooling time.

## Computational details

The computational details are thoroughly explained in the ESI† Fig. S2. Here we outline the main aspects of the methodology. All quantum-mechanical (QM) calculations were done with the complete active space self-consistent field (CASSCF) method with an active space consisting of fourteen electrons in ten orbitals and averaged over four states (SA4-CAS(14,10)). This is the same space used in ref. 25. Due to the extensive computational resources required by the dynamic calculations (up to 3 ps) repeated over three different solvents, we adopted the 3-21G basis set.^54^ Dynamics with this basis set are in quantitative agreement with the results using 6-31G* basis set reported in ref. 25 and presents a significant computational cost reduction. Moreover, since our goal is not to discuss the internal conversion of cytosine but the energy transfer to the solvent, we only need a computational level that can successfully describe the ultrafast dynamics to S_0_.

The solvent was treated *via* molecular mechanics (MM). Three solvation schemes were considered: (1) cytosine in a 20 Å sphere containing 684 Ar atoms at 10 K; (2) cytosine in a 19.2 Å sphere with 200 benzene molecules at 298 K; (3) cytosine in a 12.9 Å sphere with 300 water molecules at 298 K. The OPLS/AA force field was used for Ar, cytosine, and benzene. For Ar, standard OPLS/AA^55^ parameters were used, while for cytosine and benzene, they were obtained using the LigParGen web server.^56–58^ The TIP3P^59^ force field was used for water. The solute–solvent interaction was computed through QM/MM in an electrostatic embedding.^60^ Cytosine was in the QM region and the solvent in the MM region.

Solute–solvent (microcanonical) nonadiabatic dynamics simulations were done with QM/MM surface hopping. The hopping probabilities were computed with the decoherence-corrected^61^ fewest-switches surface hopping^62^ (DC-FSSH). The quantum integration is done with 0.025 fs using interpolated electronic quantities between classical steps of 0.5 fs. The initial conditions were generated with the hybrid Wigner-thermal protocol proposed in ref. 52. For the simulations in argon and benzene, we ran 50 trajectories each. In water, we ran 75 trajectories. The initial state was distributed over S_1_ to S_3_ according to the transition probabilities in the 5.25 ± 0.25 eV energy window.

All CASSCF calculations were done with the COLUMBUS program.^63–66^ The MM calculations were done using TINKER software.^67^ Surface hopping dynamics was performed using the NEWTON-X software^68^ interfaced with COLUMBUS and TINKER.

## Results

We used the QM/MM nonadiabatic dynamics data for cytosine in argon, benzene, and water to determine the parameters of the energy-transfer model introduced in Section Modeling the heat transfer. In this way, it was possible to link the type of chromophore–solvent interaction with the heating and cooling rates of the chromophore. The nonadiabatic mechanisms of cytosine dynamics in each solvent are discussed in ESI† Fig. S3. Here, we exclusively focus on the time constants, which are the critical pieces of information for the energy-transfer model.

**Fig. 2:**
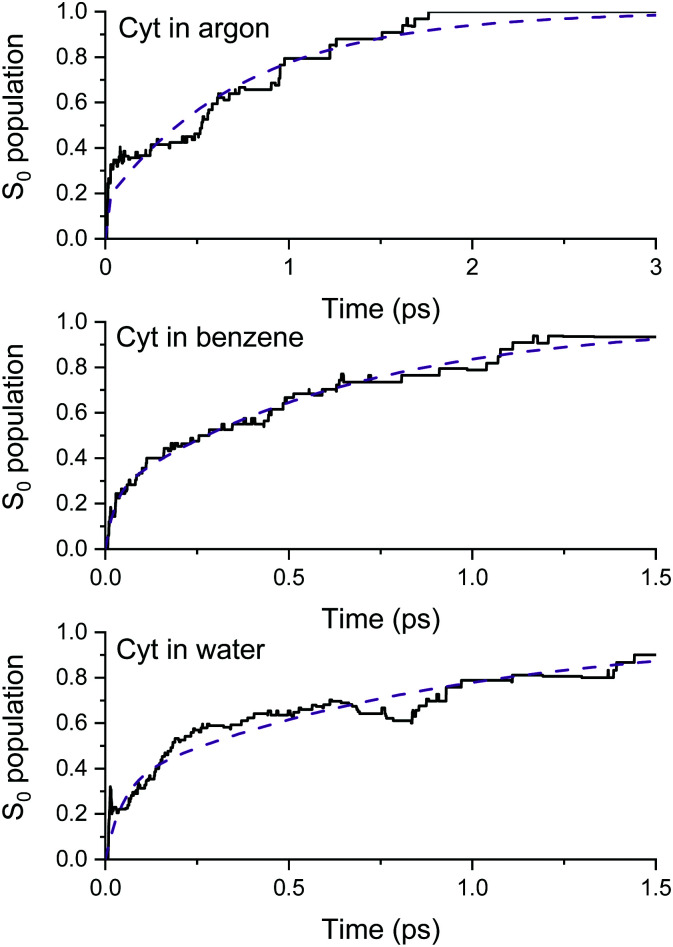
Ground-state population as a function of time simulated with surface hopping for cytosine in argon (top), benzene (middle), and water (bottom). The dashed lines are the fitting function from eqn (2).

Table 1 shows the weights (*a*_*i*_), time constants *τ*^c^_*i*_ (see eqn (3)), and cytosine's average excited-state lifetime in the three solvents and in the gas phase (from ref. 25). These results are obtained by fitting the fraction of trajectories in the ground state as a function of time with eqn (2) up to 1.0 ps in the gas phase, 3 ps in argon, 1.5 ps in benzene, and 1.5 ps in water (see Fig. 2).

**Table 1:** Weights and time constants of the two decaying channels of cytosine in the gas phase and the three solvents (eqn (2) and (3)) and average excited-state lifetime (eqn (12))

	Solvent			
	Gas phase^a^	Argon	Benzene	Water
*a*_1_	0.16	0.16	0.24	0.33
*τ* ^c^_1_ (ps)	0.013	0.0070	0.024	0.046
*a*_2_	0.84	0.84	0.76	0.67
*τ* ^c^_2_ (ps)	0.688	0.760	0.651	0.903
*τ*_H_ (ps)	0.58	0.64	0.50	0.62

a Ref. 25.

**Fig. 3:**
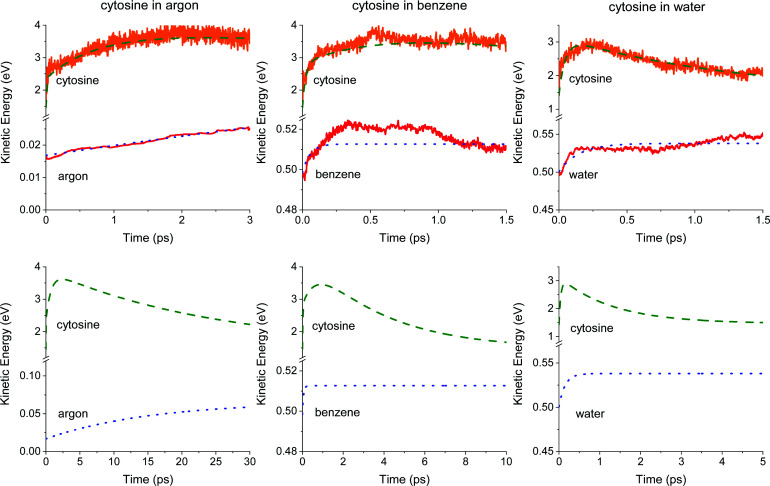
Time dependence of the mean kinetic energies of cytosine and solvents. The upper graphs show the results from the dynamics averaged over the trajectories. The dashed and dotted lines are the energy-transfer functions of eqn (6) for cytosine and eqn (4) for the solvent. These same functions are shown in the bottom graphs but over an extended time. The kinetic energy of the solvent is scaled by *N*_c_/*N*_s_, like in eqn (1).

The QM/MM mean kinetic energies of cytosine *E*_c_(*t*) and each solvent *E*_s_(*t*) are shown in Fig. 3. The results of the energy-transfer model (eqn (4) and (6)) are plotted too (*N*_c_ = 13 and *hν* = 5.25 eV). The agreement between them is outstanding. The bottom graphs in Fig. 3 show the long-timescale behavior of the energy-transfer model. The energy distribution between translational and internal degrees is examined in ESI† Fig. S4.

The parameters of the energy-transfer model describing the solvent's kinetic energy *E*_s_(*t*) are given in Table 2. Argon's kinetic energy mainly grows with a single time constant of 15.5 ps. It is much longer than the 0.64 ps excited-state lifetime (Table 1). This result implies that the energy transfer to the solvent happens mostly after cytosine returns to the ground state. In the case of water, the situation is the opposite: water's kinetic energy grows within 0.15 ps, which is much shorter than the excited-state lifetimes (0.62 ps), meaning that the energy transfer starts while cytosine is still excited. Benzene kinetic energy grows in the first 0.5 ps above the equilibrium value and converges to it from above (Fig. 3, middle-bottom). We have tracked this behavior to a mismatch between the MM-equilibrated initial conditions and the QM/MM-level dynamics. Although this artifact rendered too short *τ*^s^_1_ (0.06 ps in Table 2), it does not impact our main results, the description of *E*_c_(*t*), because *E*_c_(*t*) ≫ *E*_s_*N*_c_/*N*_s_ in eqn (1).

**Table 2:** Weights, time constants, and equilibrium constant describing the solvent kinetic energy time evolution *E*_s_ (*t*) (see eqn (4))

	Solvent		
	Argon	Benzene	Water
*N*_s_	684	2400	900
*E* ^0^_s_ (eV)	0.89	92.03	34.66
*E* ^∞^_s_ (eV)	3.46	94.64	37.25
*κ* ^s^_1_ (eV)	−2.576	−2.61	−2.59
*τ* ^s^_1_ (ps)	15.49	0.06	0.15

The energy-transfer parameters for cytosine are given in Table 3. In the three solvents, *B*_1_ ≪ *A*_*i*_, which is consequence of the much larger kinetic energy (per atom) in cytosine than in the solvent. It also implies a low level of back energy transfer, as we assumed in the model. In water, we additionally observe *A*_2_ = 0, meaning that the cytosine's heating through the second decay pathway is canceled out by its simultaneous energy transfer to the solvent.

**Table 3:** Energy-transfer parameters and cooling time, obtained from cytosine's kinetic energy *E*_c_(*t*) (see eqn (6)) in the three solvents

	Solvent		
	Argon	Benzene	Water
*E* ^0^_c_ (eV)	1.46	1.46	1.43
*E* ^∞^_c_ (eV)	1.50	1.47	1.47
*A*_1_ (eV)	−0.97	−1.37	−1.72
*A*_2_ (eV)	−1.55	−1.74	0.00
*B*_1_ (eV)	0.08	0.00	0.005
*C* (eV)	2.40	310	1.68
*τ*_C_ (ps)	24.9	3.7	1.3

The cytosine cooling time, *τ*_C_, is also given in Table 3. As expected, the energy transfer in argon is the slowest, taking about 25 ps. This time drops to about 4 ps in benzene and is merely 1.3 ps in water.

## Discussion

Our model showed that the heating of cytosine due to internal conversion occurs within about half a picosecond independently of the solvent (*τ*_H_ in Table 1). On the other hand, cytosine cooling happens within 25 ps in argon, 4 ps in benzene, and 1.3 ps in water (*τ*_C_ in Table 3). In the case of benzene and, especially, argon, their cooling times are longer than the dynamics simulations. Nevertheless, our energy-transfer model is not a simple fitting functional. It is based on physically motivated equations, parameter constraints (eqn (5) and (11)), and boundary conditions (eqn (9) and (10)), compensating for the short simulation times.

Our estimate for the cooling time of cytosine in benzene is 4 ps (Table 3). For comparison, after excitation at 261 nm, the cooling of coumarin in cyclohexane is about 10 ps, as measured with time-resolved fluorescence spectroscopy.^69^ The chromophore-solvent interaction between cytosine and benzene should be stronger than that between coumarin and cyclohexane (as the π–π interaction is absent in the latter), explaining the slightly shorter time for cytosine.

Zhang *et al.*,^70^ using UV-pump/broadband-mid-IR-probe transient absorption spectra, associated the cooling time of hot purine derivatives to the directionality of their H-bonds, showing that molecules that have more N–H bonds have shorter cooling times. They proposed that N–H(D) bond donation is responsible for rapid energy disposal to water *via* direct coupling of high-frequency solute–solvent modes. Thus, the cooling times decrease in the order caffeine (7.7 ps) > theophylline (5.1 ps) > hypoxanthine (3 ps) due to the larger number of N–H bonds.^70^ Our value of 1.3 ps obtained for cytosine in water (Table 3) fits very well in this sequence, as our chromophore makes three N–H bonds, while hypoxanthine has two. Our cooling time is consistent with the vibrational cooling of 2 to 3 ps of photoacid pyranine^71^ and 2.4 ps of 9-methyl-adenine,^72^ both in water. Foremost, it is consistent with the vibrational cooling of cytosine in water, which is reported as ∼2.9 ps^23,31^ and ∼4.0 ps.^73^ Indeed, it has been shown that hydrogen bonds between nucleobase monomers and solvent strongly enhance vibrational cooling.^22,31^ The importance of H-bonds for a faster solute–solvent vibrational energy relaxation has also been pointed out by Pigliucci *et al.*,^74^ who identified that such process is enhanced for solutes (substituted perylenes) bonded to alcohols.

Cytosine's kinetic energy reaches a maximum after 2.38 ps in argon, 0.95 ps in benzene, and 0.16 ps in water, as we can see in Fig. 3. This time represents a transient equilibrium between the energy that the internal conversion transfers to cytosine's vibrational modes and the energy cytosine transfers to the solvent. Interestingly, this transient equilibrium in water happens before the internal conversion (0.62 ps), meaning cytosine starts to cool down while still photoexcited. The outcome for water is consistent with the findings of ref. 74, which concluded that both intra- and intermolecular (solute–solvent) vibrational energy redistribution processes occur, at least partially, on similar timescales.

We do not expect that the modest computational levels employed in this work will be the last word in the description of cytosine-solvent transfer times, although the analysis above shows a semi-quantitative agreement with previous results. Nevertheless, a remarkable feature of our model is that it is independent of the Hamiltonian, and it can be employed with more advanced methods,^75,76^ as long as nonadiabatic dynamics is affordable.

## Conclusion

In this work, we developed an energy-transfer model to treat the heating and the cooling of a solute in different types of solvents due to internal conversion after an electronic excitation. It captures the primary causes of heating and cooling of a chromophore in solution after photoexcitation, the vibrational energy released after internal conversion, and the energy transfer to the solvent. The model is inspired by the one proposed by Balevičius Jr *et al.*,^1^ but it goes beyond by incorporating nonadiabatic information, allowing equal footing treatment of energy transfer from excited and ground states to the solvent. The model is fed by standard quantities available in nonadiabatic dynamics—the ground-state population and kinetic energies (of solute and solvent) as a function of time—and it allows determining heating and cooling times. Therefore, the proposed model is expected to be suitable for any type of nonadiabatic molecular dynamics simulation method. The use of physically motivated equations, parameter constraints, and boundary conditions reduces the need for long-timescale propagation for finding the time constants.

We have applied this energy-transfer model to a chromophore embedded into three types of solvents, argon, benzene, and water, spanning an extensive range of interactions. Due to its complete and ultrafast (sub-picosecond) internal conversion, we adopted cytosine as a prototypical chromophore. Using data from surface hopping dynamics with QM/MM, our model predicts that cytosine cools down within 25 ps in argon, 4 ps in benzene, and 1.3 ps water.

Our initial goal in this work was to estimate the chromophore-to-solvent heat transfer times. These results for cytosine in argon, benzene, and water may be taken as a qualitative indication of the orders of magnitude of the cooling time of other organic chromophores in similar solvents. Nevertheless, the development of an energy-transfer model that can be directly employed with results from any nonadiabatic dynamics simulations goes much beyond that initial goal and delivers a new protocol to analyze energy transfer in complex environments. Currently the model is being extended to also take into account the heat transfer between strongly coupled subsystems (*e.g.* through a covalent bond), as in the case of nucleosides.

## Data availability

The raw QM/MM input and output data are available for download at https://doi.org/10.5281/zenodo.5883863.

## Author contributions

M. B., E. V. and S. A. M. conceived the computational studies and developed the theoretical model for energy transfer. E. V. and S. A. M. conducted the computational studies. All authors contributed in interpreting the data and writing the manuscript.

## Conflicts of interest

There are no conflicts to declare.

## Supplementary Material

CP-024-D2CP00686C-s001
