# Correction: Modeling the heating and cooling of a chromophore after photoexcitation

**DOI:** 10.1039/d2cp90104h

**Published:** 2022-06-15

**Authors:** Elizete Ventura, Silmar Andrade do Monte, Mariana T. do Casal, Max Pinheiro, Josene Maria Toldo, Mario Barbatti

**Affiliations:** Universidade Federal da Paraíba 58059-900 João Pessoa-PB Brazil elizete@quimica.ufpb.br silmar@quimica.ufpb.br; Aix Marseille University, CNRS, ICR Marseille France mario.barbatti@univ-amu.fr https://barbatti.org; Institut Universitaire de France 75231 Paris France

## Abstract

Correction for ‘Modeling the heating and cooling of a chromophore after photoexcitation’ by Elizete Ventura *et al.*, *Phys. Chem. Chem. Phys.*, 2022, **24**, 9403–9410, https://doi.org/10.1039/D2CP00686C.

The published version of this article contained errors in part of the funding information in the Acknowledgements. The corrected funding information for M. T. do C., J. M. T. and M. B. is as follows: M. T. do C., J. M. T. and M. B. have received funding from the European Union’s Horizon 2020 research and innovation programme under the grant agreement no. 828753.

The Royal Society of Chemistry apologises for these errors and any consequent inconvenience to authors and readers.

## Supplementary Material

