# Supplementary material for: Modeling the heating and cooling of a chromophore after photoexcitation
Source: Phys Chem Chem Phys. 2022 Apr 5;24(16):9403–10. doi: 10.1039/d2cp00686c (PMC9020442; doi:10.1039/d2cp00686c)
Supplement: CP-024-D2CP00686C-s001 [file CP-024-D2CP00686C-s001.pdf]

Electronic Supplementary Material (ESI) for Physical Chemistry Chemical Physics.
This journal is © the Owner Societies 2022

ELECTRONIC SUPPLEMENTARY INFORMATION

Modeling the heating and cooling of a chromophore after photoexcitation

Elizete Ventura,*[a] Silmar Andrade do Monte,*[a] Mariana Telles do Casal,[b] Max Pinheiro Jr.,[b] Josene Maria Toldo,[b] Mario Barbatti *[b,c]

* Corresponding authors
E-mail (EV): elizete@quimica.ufpb.br
Email (SAM): silmar@quimica.ufpb.br
E-mail (MB): mario.barbatti@univ-amu.fr; Website: barbatti.org
[1] Universidade Federal da Paraíba, 58059-900, João Pessoa-PB, Brazil
[1] Aix Marseille University, CNRS, ICR, Marseille, France
[1]*Institut Universitaire de France, 75231 Paris, France*

Table of Contents

S1. KINETIC ENERGY PARTITION 2

S2. COMPUTATIONAL DETAILS 3

A. QM CALCULATIONS 3
B. MM AND QM/MM CALCULATIONS 4
C. INITIAL CONDITIONS FOR DYNAMICS 5
D. NONADIABATIC DYNAMICS 6

S3. DYNAMICS OF CYTOSINE 7

S4. INTERNAL AND TRANSLATIONAL ENERGIES 9

S5. REFERENCES 10

S1. Kinetic energy partition

When $t \to \infty$, the photon energy $h\nu$ should distribute among all degrees of freedom. If we assume equipartition, each degree should receive

$$\varepsilon = \frac{h\nu}{3\left(N_c + N_s\right)}, \tag{1}$$

where N_c is the number of atoms in the chromophore and $N_s = N_{s,m} \times N_{s,a}$ is the total number of solvent atoms, considering that there are $N_{s,m}$ solvent molecules with $N_{a,m}$ atoms each.

We assume that the vibrational degrees of each molecule are approximately harmonic. Thus, the virial partition between kinetic and energy tells that the kinetic contribution of each vibrational degree is

$$\varepsilon_{kin}^{v} = \frac{\varepsilon}{2}. \tag{2}$$

In the case of the translational and rotational modes, we may have two situations. If the molecule feels the neighbours' potential, we once more assume a virial partition and attribute $\varepsilon / 2$ to the kinetic contribution of the mode. However, if the molecule is in a near-ideal-gas environment, the kinetic contribution of each mode is ε. Thus, each translational and rotational mode's kinetics is

$$\varepsilon_{kin}^{t,r} = \gamma\varepsilon, \tag{3}$$

where

$$\gamma = \begin{cases} 1/2 & \text{for crystals and H-bonded systems} \\ 1 & \text{for ideal gas} \end{cases} \tag{4}$$

The photon contribution for the equilibrium kinetic energies of the chromophore is

$$\begin{aligned} K_c &= \left(6\gamma + \frac{1}{2}\left(3N_c - 6\right)\right)\varepsilon \\ &= 2\left(\frac{N_c}{4} + \gamma - \frac{1}{2}\right)\frac{h\nu}{\left(N_c + N_s\right)}, \end{aligned} \tag{5}$$

and for the solvent

$$K_s = \left(6\gamma + \frac{1}{2}\left(3N_{a,m} - 6\right)\right) N_{s,m}\varepsilon$$
$$= 2N_{s,m}\left(\frac{N_{a,m}}{4} + \gamma - \frac{1}{2}\right)\frac{h\nu}{\left(N_c + N_s\right)}. \quad (6)$$

The equilibrium kinetic energy of the chromophore $(x = c)$ and solvent $(x = s)$ is

$$E_x^\infty = E_x^0 + K_x \quad (7)$$

In all three cases treated in our paper—argon matrix, liquid benzene, and water—$\gamma = 2$. Thus,

$$E_c^\infty = E_c^0 + \frac{N_c}{2\left(N_c + N_s\right)} h\nu,$$
$$E_s^\infty = E_s^0 + \frac{N_s}{2\left(N_c + N_s\right)} h\nu. \quad (8)$$

S2. Computational details

A. QM Calculations

The ground state geometry of cytosine was optimized at Complete Active Space Self-consistent Field (CASSCF) level. The complete active space consists of fourteen electrons in ten orbitals, i.e., a CAS(14,10). These ten orbitals at the ground state minimum geometry are three n, four π, and three π* orbitals, described in detail in ref.[1] The first four singlet states were averaged with equal weights. A normal-mode analysis was also performed to confirm that the optimized geometry is a minimum. The geometry optimization and normal-mode calculation were performed using analytical gradient techniques.[2-5] Frequency calculations were performed via finite differences from analytic gradients. All CASSCF calculations were done with the COLUMBUS program.[6-9] The atomic orbitals (AO) integrals and their gradient integrals used by COLUMBUS were computed with the corresponding modules taken from the DALTON program.[10]

Due to the extensive computational resources required by the dynamic calculations (up to 3ps) repeated over three different solvents, we adopted the 3-21G basis set.[11] Dynamics simulations with this basis set are in semi-quantitative agreement with the results using the 6-31G* basis set reported in ref.[1] and present a significant computational cost reduction. Moreover, since our goal is not to discuss the internal conversion of cytosine but the energy transfer to the solvent, we only need a computational level that can successfully describe the ultrafast dynamics to S_0.

B. MM and QM/MM calculations

To build the cluster with the cytosine molecule inside an Ar matrix, a similar procedure as described in ref.[12] was used. First, the Ar matrix was built by increasing the unit cell of the experimental X-ray structure[13] to dimensions 50 x 50 x 50 Å (yielding 1372 Ar atoms), using the vegaZZ software.[14] The same program was used to insert the cytosine molecule inside the cavity. To ensure that the cavity was large enough to accommodate the solute, the default maximum overlap (0.80Å) between solute and neighboring Ar atoms in the matrix was applied, leading to a removal of four Ar atoms from the center of the crystal matrix. Then, a sphere of 20 Å containing the cytosine and 684 Ar atoms was built for the subsequent equilibration (described later). This procedure was carried out only once for one of the selected Wigner structures of cytosine. The remaining clusters were generated by replacing the cytosine structure with each of the other selected cytosine geometries generated by the Wigner distribution.

Spherical clusters of cytosine in benzene and water were built using the PACKMOL package,[15] with radii of 19.2 Å and 12.9 Å, respectively. The number of solvent molecules (200 and 300 for benzene and water, respectively) was chosen based on their densities at 298 K, 0.876 (benzene) and 0.997 (water) g/cm^3.[16] As for the Ar cluster, such procedure was done only once, using one of the Wigner structures generated for cytosine (see Section C). For the remaining ones, the cytosine structure was replaced accordingly.

All MM calculations were done using TINKER software.[17] Once the cluster structures were obtained, they were thermalized at the MM level using the NVT ensemble, keeping the cytosine frozen. A temperature of 10 K was used for the Ar cluster, while 298

K was used for benzene and water clusters. The equilibration times varied from 5 to 150 ps, depending on the solvent. The Ar clusters required larger equilibration times. The "wall" keyword in TINKER was used during the equilibration to maintain droplet boundary conditions, preventing solvent atoms from moving outside the defined spherical radius. The OPLS/AA force field was used for Ar, cytosine, and benzene. For Ar, standard OPLS/AA[18] parameters were used, while for cytosine and benzene, they were obtained using the LigParGen web server.[19-21] The TIP3P[22] force field was used for water. Once the clusters were equilibrated, the initial coordinates of solvent molecules and solute (this latter generated by the Wigner distribution) needed for nonadiabatic QM/MM dynamics were defined. The initial velocities, also required for the initial conditions, were taken from the Wigner distribution for cytosine and MM equilibration for solvent molecules. This procedure, proposed in ref.[23], prevents the nuclei of the solute from having energies below the zero point but ensures that the solvent is at the correct temperature.

The solute-solvent interaction was computed through QM/MM in an electrostatic embedding.[24] Cytosine was in the QM and the solvent in the MM regions.

C. Initial conditions for dynamics

Initial geometries and velocities for the QM region (cytosine) were generated by a Wigner distribution, where each nuclear coordinate is treated as a quantum harmonic oscillator in the ground state. Initial conditions were randomly selected in terms of transition probabilities into the first three excited states and restricted to the same spectral window used in ref.[1], that is, 5.25±0.25 eV. Besides this energy restriction, initial conditions were randomly selected in terms of transition probabilities into the three excited states. From the original 1500 possible initial conditions (500 random points starting in each of the three excited states), this procedure yielded 79 initial conditions, with 2, 47, and 30 starting in S_1, S_2, and S_3, respectively. We chose a reduced number of trajectories keeping this proportion for the subsequent QM/MM calculations (see Section D).

D. Nonadiabatic dynamics

Nonadiabatic dynamics was simulated with QM/MM surface hopping. The hopping probabilities were computed with the decoherence-corrected[25] fewest-switches surface hopping[26](DC-FSSH) algorithm. The quantum integration was done with 0.025 fs using interpolated electronic quantities between classical steps of 0.5 fs. Time-dependent electronic coefficients were adjusted for decoherence with the simplified decay of mixing method.[25] The decoherence parameter was set to 0.1 au. In the case of hopping, the momentum excess is adjusted in the direction of the nonadiabatic coupling vectors, while in the case of frustrated hopping, the momentum is kept constant. Additional details concerning the integration of classical and quantum equations are given in ref[27]. Whenever a given trajectory remained for at least 50 fs in the ground state, it was restarted adiabatically in this state. Anelastic-collision spherical boundary was applied to keep the density constant. The sphere is centered at cytosine's center of mass with the cluster radius. Surface hopping dynamics was performed using the NEWTON-X software[28] interfaced with COLUMBUS and TINKER.[23]

Table 1 Number of trajectories starting in each initial state. 18 of the 30 trajectories in argon were extended to 3 ps.

Solvent	Set	S_1	S_2	S_3
**Argon**	50	1	30	19
**Benzene**	50	1	30	19
**Water**	75	2	45	28

As discussed in Section C, we have yielded 79 initial conditions, 2 starting in S_1, 47 in S_2, and 30 in S_3. For the simulations in argon, we ran 50 trajectories (1 starting in S_1, 30 in S_2, and 19 in S_3). In benzene, we also ran 50 trajectories with the same distribution of initial states. In water, we ran 75 trajectories (2 starting in S_1, 45 in S_2, and 28 in S_3). The number of trajectories in each initial state is summarized in Table 1.

In argon, 30 trajectories run at least 1.8 ps, and 18 ended at 3 ps. In benzene and water, 30 trajectories of each solvent ran at least 1.5 ps. In argon, the heat-transfer model

was fitted with data up to 3 ps. In benzene and water, the fitting was done with data up to 1.5 ps.

S3. Dynamics of cytosine

As expected, the time constants and the excited-state lifetime of cytosine in argon matrix and benzene are close to that obtained in the gas phase.[1] However, in water, the corresponding time constants are considerably larger, although the average excited-state lifetime (0.62ps) is also close to those obtained in Ar and benzene. According to previous surface hopping simulations in the gas phase,[1] the larger time constant could be interpreted as a decay channel mainly through a semi-planar conical intersection along the $n_O\pi^*$ surface. In fact, from the fraction of trajectories that ended up adiabatically in S_0, i.e., the ones which were restarted as adiabatic dynamics in the S_0 after the S_1/S_0 crossing (90, 78, and 76% in Ar, benzene, and water, respectively), 97% (Ar), 85% (benzene), and 100 % (water) decayed through this semi-planar conical intersection.

The weight and the time constant associated with the ultrafast decay channel (< 50 fs) increase in the following order: argon < benzene < water. These two results explain the average excited-state lifetime of cytosine in water being close to those in the other two solvents, despite its significantly larger τ_2^c value. The second most important conical intersection associated with the $S_1 \rightarrow S_0$ decay is the C6-puckered,[1] also reported as an ethylenic conical intersection, which involves the decay of the $\pi\pi^*$ state.[29-30] It was observed in 4% of the trajectories in Ar and 10% of the trajectories in benzene. In this last solvent, 3% of the trajectories decayed through either the oop-NH_2 (associated with the decay of $n_N\pi^*$ state) or oop-O conical intersections.[1]

In water, although $n\pi^*$ states are expected to be destabilized, several works have shown that they play an essential role in the nonradiative decay of cytosine.[31-33] Time-resolved fluorescence (TRF)[34-35] and time-resolved infrared (TRIR)[36-37] experiments report only the involvement of $\pi\pi^*$ state, which decays with the time constants of ~0.2 and 1.0 –1.5 ps. On the other hand, transient electronic absorption spectroscopy (TEAS)[32, 35, 38] also revealed that part of the population would be trapped in a long-lived dark state and decay through an $n\pi^*/S_0$ conical intersection with longer time constants (7.7– 12 ps).

Computational simulations including explicit water molecules support the involvement of the nπ* state.[31, 33]

Although the ultrafast time constants of cytosine in water are often linked to the decay of ππ* state, the agreement between experimental and our theoretical results is reasonable, considering the electronic-structure level and the number of trajectories in our simulations. Therefore, because our simulations do not include explicit water molecules in the QM part, it is natural that our results resemble the ones in the gas phase,[1] since quantum-mechanical interactions, such as water-chromophore electron transfer,[33] are not captured at this level of theory. Nevertheless, the strength of the cytosine-water hydrogen bonds is correctly predicted by our QM/MM partition, which is enough to ensure the energy flow between the subsystems.

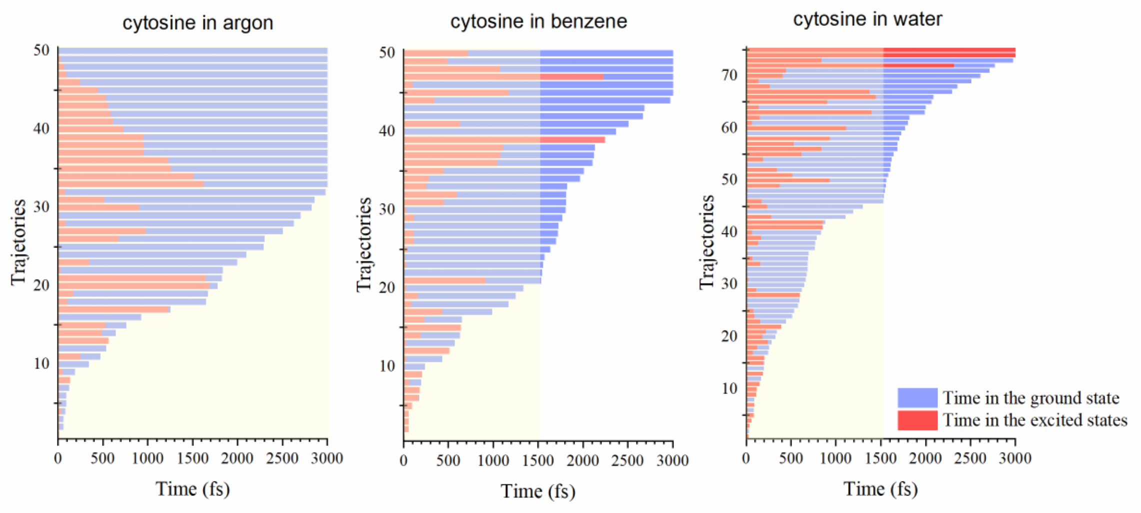

Figure 1. Total time reached by each trajectory of the three QM/MM systems studied in this work. Time intervals in the ground and excited states are indicated in blue and red, respectively. The shaded regions indicate the trajectories considered in the population analysis and the model's fitting (see Figures 2 and 3 of the manuscript) for each solvent. This region includes trajectories up to 3.0 ps for cytosine in Ar and 1.5 ps for cytosine in benzene and water.

It is worth mentioning that a fraction of trajectories (6 to 20%, depending on the system) remained in the excited states, similar to earlier surface hopping simulation in the

gas phase.[1] This allows an alternative interpretation, using a tri-exponential fitting, where part of the population would decay with longer time constants.

Figure 1 shows additional details concerning the time and the states (ground or excited) reached by each trajectory of the three QM/MM systems.

S4. Internal and translational energies

We also wanted to determine to what extent the internal vibrational degrees of freedom of the solvent molecules take part in the heat transfer. To figure this out, we partitioned the total kinetic energy of the solvent (E_s) into translational $(E_{s,t})$ and internal $(E_{s,\mathrm{i}} = E_s - E_{s,t})$ contributions for the simulations in benzene and water. The time dependence of the $E_{s,i} / E_s$ ratio is depicted in Figure 2.

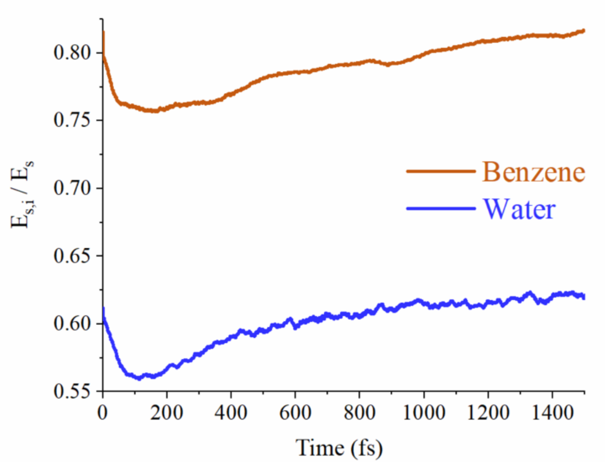

Figure 2. Time dependence of the $E_{s,i} / E_s$ ratio computed for benzene and water.

Each solvent molecule has three translational and 3*N*–3 internal (rotational plus vibrational) degrees of freedom, where *N* is the number of atoms. Assuming equipartition of kinetic energy among all 3*N* degrees of freedom, one has $E_{s,i} / E_s = (N-1)/N$. Thus, the expected ratios for benzene and water are ~0.9 and 0.7, respectively, which are in good agreement with the average values in Figure 2, ~0.8 and 0.6.

The initial reduction of the ratios shown in Figure 2 is an artifact due to the MM equilibration performed on the clusters before they were submitted to the nonadiabatic QM/MM dynamics. The initial decrease can be due to a relaxation of the system under the influence of the new QM/MM environment. It is expected that with an initial equilibration at the QM/MM level,[12] the ratio should show a smaller variation.

S5. References

1. Barbatti, M.; Aquino, A. J.; Szymczak, J. J.; Nachtigallova, D.; Lischka, H., Photodynamical simulations of cytosine: characterization of the ultrafast bi-exponential UV deactivation. *Phys Chem Chem Phys* **2011**, *13* (13), 6145-55.

2. Shepard, R.; Lischka, H.; Szalay, P. G.; Kovar, T.; Ernzerhof, M., A general multireference configuration interaction gradient program. *J. Chem. Phys.* **1992**, *96* (3), 2085-2098.

3. Shepard, R., The Analytic Gradient Method For Configuration Interaction Wave Functions. In *Modern Electronic Structure Theory*, World Scientific Publishing Company: 1995; pp 345-458.

4. Shepard, R., Geometrical energy derivative evaluation with MRCI wave functions. *Int. J. Quantum Chem.* **1987**, *31* (1), 33-44.

5. Lischka, H.; Dallos, M.; Shepard, R., Analytic MRCI gradient for excited states: formalism and application to the n-π∗ valence- and n-(3s,3p) Rydberg states of formaldehyde. *Mol. Phys.* **2002**, *100* (11), 1647-1658.

6. Lischka, H.; Shepard, R.; Shavitt, I.; Pitzer, R. M.; Dallos M.; Müller, T.; Szalay P. G.; Brown, F. B.; Ahlrichs, R.; Böhm, H. J.; Chang, A.; Comeau, D. C.; Gdanitz, R.; Dachsel, H.; Ehrhardt, C.; Ernzerhof, M.; Höchtl, P.; Irle, S.; Kedziora, G.; Kovar, T.; Parasuk V.; Pepper, M. J. M.; Scharf, P.; Schiffer, H.; Schindler, M.; Schüler, M.; Seth M.; Stahlberg, E. A.; Zhao, J.-G.; Yabushita, S.; Zhang, Z.; Barbatti, M.; Matsika, S.; Schuurmann, M.; Yarkony, D. R.; S. R. Brozell; Beck, E. V.; Blaudeau, J.-P.; Ruckenbauer, M.; Sellner, B.; Plasser, F.; Szymczak, J. J.; Spada, R. F. K.; Das, A., *COLUMBUS, an ab initio electronic structure program, release 7.0 (2017).*

7. Lischka, H.; Shepard, R.; Pitzer, R. M.; Shavitt, I.; Dallos, M.; Müller, T.; Szalay, P. G.; Seth, M.; Kedziora, G. S.; Yabushita, S.; Zhang, Z., High-level multireference methods

in the quantum-chemistry program system COLUMBUS: Analytic MR-CISD and MR-AQCC gradients and MR-AQCC-LRT for excited states, GUGA spin–orbit CI and parallel CI density. *Phys. Chem. Chem. Phys.* **2001**, *3* (5), 664-673.

8. Lischka, H.; Shepard, R.; Brown, F. B.; Shavitt, I., New implementation of the graphical unitary group approach for multireference direct configuration interaction calculations. *Int. J. Quantum Chem.* **1981**, *20* (S15), 91-100.

9. Lischka, H.; Shepard, R.; Müller, T.; Szalay, P. G.; Pitzer, R. M.; Aquino, A. J. A.; Araújo do Nascimento, M. M.; Barbatti, M.; Belcher, L. T.; Blaudeau, J.-P.; Borges, I.; Brozell, S. R.; Carter, E. A.; Das, A.; Gidofalvi, G.; González, L.; Hase, W. L.; Kedziora, G.; Kertesz, M.; Kossoski, F.; Machado, F. B. C.; Matsika, S.; do Monte, S. A.; Nachtigallová, D.; Nieman, R.; Oppel, M.; Parish, C. A.; Plasser, F.; Spada, R. F. K.; Stahlberg, E. A.; Ventura, E.; Yarkony, D. R.; Zhang, Z., The generality of the GUGA MRCI approach in COLUMBUS for treating complex quantum chemistry. *J. Chem. Phys.* **2020**, *152* (13), 134110.

10. Helgaker, T.; Jensen, H. J. A.; Jørgensen, P.; Olsen, J.; Ruud, K.; Ågren, H.; Andersen, T.; Bak, K. L.; V. Bakken; Christiansen, O.; P. Dahle; Dalskov, E. K.; Enevoldsen, T.; Heiberg, H.; Hettema, H.; Jonsson, D.; Kirpekar, S.; Kobayashi, R.; Koch, H.; Mikkelsen, K. V.; Norman, P.; Packer, M. J.; Saue, T.; Taylor, P. R.; Vahtras, O., *DALTON, an ab initio electronic structure program, Release 1.0, 1997*.

11. Binkley, J. S.; Pople, J. A.; Hehre, W. J., Self-consistent molecular orbital methods. 21. Small split-valence basis sets for first-row elements. *J. Am. Chem. Soc.* **1980**, *102* (3), 939-947.

12. Eckert-Maksić, M.; Vazdar, M.; Ruckenbauer, M.; Barbatti, M.; Müller, T.; Lischka, H., Matrix-controlled photofragmentation of formamide: dynamics simulation in argon by nonadiabatic QM/MM method. *Phys. Chem. Chem. Phys.* **2010**, *12* (39), 12719-12726.

13. Barrett, C. S.; Meyer, L., X-Ray Diffraction Study of Solid Argon. *J. Chem. Phys.* **1964**, *41* (4), 1078-1081.

14. Pedretti, A.; Villa, L.; Vistoli, G., VEGA: a versatile program to convert, handle and visualize molecular structure on Windows-based PCs. *J. Mol. Graph. Model.* **2002**, *21* (1), 47-49.

15. Martínez, L.; Andrade, R.; Birgin, E. G.; Martínez, J. M., PACKMOL: A package for building initial configurations for molecular dynamics simulations. *J. Comput. Chem.* **2009**, *30* (13), 2157-2164.

16. *CRC Handbook of Chemistry and Physics*. 97th Edition ed.; CRC Press, 2017. Taylor & Francis Group, Boca Raton, FL: 2017.

17. Rackers, J. A.; Wang, Z.; Lu, C.; Laury, M. L.; Lagardère, L.; Schnieders, M. J.; Piquemal, J.-P.; Ren, P.; Ponder, J. W., Tinker 8: Software Tools for Molecular Design. *J. Chem. Theory Comput.* **2018**, *14* (10), 5273-5289.

18. Jorgensen, W. L.; Maxwell, D. S.; Tirado-Rives, J., Development and Testing of the OPLS All-Atom Force Field on Conformational Energetics and Properties of Organic Liquids. *J. Am. Chem. Soc.* **1996**, *118* (45), 11225-11236.

19. Jorgensen, W. L.; Tirado-Rives, J., Potential energy functions for atomic-level simulations of water and organic and biomolecular systems. *Proc. Natl. Acad. Sci. USA* **2005**, *102* (19), 6665-6670.

20. Dodda, L. S.; Vilseck, J. Z.; Tirado-Rives, J.; Jorgensen, W. L., 1.14*CM1A-LBCC: Localized Bond-Charge Corrected CM1A Charges for Condensed-Phase Simulations. *J. Phys. Chem. B* **2017**, *121* (15), 3864-3870.

21. Dodda, L. S.; Cabeza de Vaca, I.; Tirado-Rives, J.; Jorgensen, W. L., LigParGen web server: an automatic OPLS-AA parameter generator for organic ligands. *Nucleic Acids Res.* **2017**, *45* (W1), W331-W336.

22. Dang, L. X.; Pettitt, B. M., Simple intramolecular model potentials for water. *J. Phys. Chem.* **1987**, *91*, 3349-3354.

23. Ruckenbauer, M.; Barbatti, M.; Müller, T.; Lischka, H., Nonadiabatic Excited-State Dynamics with Hybrid ab Initio Quantum-Mechanical/Molecular-Mechanical Methods: Solvation of the Pentadieniminium Cation in Apolar Media. *J. Phys. Chem. A* **2010**, *114* (25), 6757-6765.

24. Senn, H. M.; Thiel, W., QM/MM Methods for Biomolecular Systems. *Angewandte Chemie, International Edition in English* **2009**, *48* (7), 1198-1229.

25. Granucci, G.; Persico, M., Critical appraisal of the fewest switches algorithm for surface hopping. *J Chem Phys* **2007**, *126* (13), 134114.

26. Tully, J. C., Molecular dynamics with electronic transitions. *J. Chem. Phys.* **1990**, *93* (2), 1061-1071.

27. Ruckenbauer, M.; Barbatti, M.; Müller, T.; Lischka, H., Nonadiabatic Photodynamics of a Retinal Model in Polar and Nonpolar Environment. *J. Phys. Chem. A* **2013**, *117* (13), 2790-2799.

28. Barbatti, M.; Ruckenbauer, M.; Plasser, F.; Pittner, J.; Granucci, G.; Persico, M.; Lischka, H., Newton-X: a surface-hopping program for nonadiabatic molecular dynamics. *WIREs Computational Molecular Science* **2014**, *4* (1), 26-33.

29. Hudock, H. R.; Martínez, T. J., Excited-State Dynamics of Cytosine Reveal Multiple Intrinsic Subpicosecond Pathways. *ChemPhysChem* **2008**, *9* (17), 2486-2490.

30. Blancafort, L., Energetics of Cytosine Singlet Excited-State Decay Paths—A Difficult Case for CASSCF and CASPT2†. *Photochem. Photobiol.* **2007**, *83* (3), 603-610.

31. Blancafort, L.; Migani, A., Water effect on the excited-state decay paths of singlet excited cytosine. *J. Photochem. Photobiol. A* **2007**, *190* (2-3), 283-289.

32. Hare, P. M.; Crespo-Hernández, C. E.; Kohler, B., Internal conversion to the electronic ground state occurs via two distinct pathways for pyrimidine bases in aqueous solution. *Proceedings of the National Academy of Sciences* **2007**, *104* (2), 435.

33. Szabla, R.; Kruse, H.; Šponer, J.; Góra, R. W., Water–chromophore electron transfer determines the photochemistry of cytosine and cytidine. *Phys. Chem. Chem. Phys.* **2017**, *19* (27), 17531-17537.

34. Sharonov, A.; Gustavsson, T.; Carré, V.; Renault, E.; Markovitsi, D., Cytosine excited state dynamics studied by femtosecond fluorescence upconversion and transient absorption spectroscopy. *Chem. Phys. Lett.* **2003**, *380* (1), 173-180.

35. Ma, C.; Cheng, C. C.-W.; Chan, C. T.-L.; Chan, R. C.-T.; Kwok, W.-M., Remarkable effects of solvent and substitution on the photo-dynamics of cytosine: a femtosecond broadband time-resolved fluorescence and transient absorption study. *Phys. Chem. Chem. Phys.* **2015**, *17* (29), 19045-19057.

36. Quinn, S.; Doorley, G. W.; Watson, G. W.; Cowan, A. J.; George, M. W.; Parker, A. W.; Ronayne, K. L.; Towrie, M.; Kelly, J. M., Ultrafast IR spectroscopy of the short-lived transients formed by UV excitation of cytosine derivatives. *Chem. Commun.* **2007**, (21), 2130-2132.

37. Keane, P. M.; Wojdyla, M.; Doorley, G. W.; Watson, G. W.; Clark, I. P.; Greetham, G. M.; Parker, A. W.; Towrie, M.; Kelly, J. M.; Quinn, S. J., A Comparative Picosecond Transient Infrared Study of 1-Methylcytosine and 5′-dCMP That Sheds Further Light on the Excited States of Cytosine Derivatives. *J. Am. Chem. Soc.* **2011**, *133* (12), 4212-4215.

38. Malone, R. J.; Miller, A. M.; Kohler, B. In *Singlet Excited-state Lifetimes of Cytosine Derivatives Measured by Femtosecond Transient Absorption*¶, Photochem. Photobiol., 2003.
